# The collapse of the maternity workforce in a low-birthrate, aging society: suggestions for training midwives and improving systems

**DOI:** 10.4069/whn.2025.03.14

**Published:** 2025-03-28

**Authors:** Yunmi Kim, Sohee Che

**Affiliations:** 1 College of Nursing, Gachon University, Incheon, Korea; 2 Department of Global Phenomena Research, National Museum of Ethnology, Japan

## Introduction

Low birth rates in Korea and other East Asian countries have been a longstanding issue. In 2023, the total fertility rate was 0.72 in Korea, 0.77 in Hong Kong, 0.87 in Taiwan, 0.97 in Singapore, and 1.26 in Japan [1]. The total fertility rate, defined as the average number of children a woman is expected to have during her lifetime, is rapidly declining in Asia [1]. In Korea, the rate fell from 1.21 in 2014 to 0.75 in 2024, a decrease of 0.46 over 10 years [2].

Due to the declining birth rate, the maternity workforce has significantly diminished in Korea. In 2024, 112 obstetrician-gynecologists were certified, but only 13 were certified in 2025 because of conflicts between physicians and the government. Moreover, most of these professionals chose to specialize in gynecology rather than obstetrics, an area of essential medical care [3,4]. In 2025, Seoul National University Hospital announced a recruitment plan for 12 obstetrics and gynecology fellows, yet not a single fellow applied [5]. The current Medical Service Act restricts midwifery training to training hospitals associated with obstetrics and gynecology or pediatrics. Despite the urgent need for midwifery training, the limited number of training hospitals for obstetric residents fails to meet the demand for a robust maternity workforce. Consequently, as in many other countries, midwifery training should be conducted in dedicated educational institutions. This paper highlights the necessity of amending the Medical Service Act and introduces Japan’s midwifery system as a model to address the maternity workforce shortage while proposing directions for improvement in Korea.

## Midwifery system in Japan

Japan, a neighboring country, encountered a low birth rate and aging population earlier and implemented strategies to ease the transition to such a society. In 2006, Japan introduced a policy to increase the number of medical students, and since 2015, the number of individuals passing the medical licensing exam has steadily risen. However, a comparison between 2010 and 2020 reveals that although the overall number of physicians employed in hospitals increased, the number of obstetricians declined [6,7].

Japan also confronted a significant rise in medical costs due to a shrinking labor force, an aging population, and a persistently low birth rate. To mitigate these issues, the country sought strategies to lower labor costs and reduce medical fees. In light of Japan’s cultural emphasis on vaginal birth and tradition, promoting normal vaginal deliveries conducted by midwives proved a viable option and has contributed positively to maintaining maternal and child health indicators [8].

Although the number of physicians in Japan has increased, a shortage of obstetricians in clinic-level institutions outside hospitals persists. As a result, deliveries have become concentrated in hospitals, where obstetricians face excessive workloads and on-call duties exceeding 30 hours. In response, a consensus emerged among physicians that midwives should manage normal vaginal deliveries. Accordingly, the Ministry of Health, Labour and Welfare launched the “Midwife Utilization Promotion Project,” aiming to “establish a system that meets the diverse needs of pregnant women and ensures safe and comfortable delivery,” while explicitly stating that the initiative would also “relieve the burden of obstetricians” [9].

Following a 2017 notification from Japan’s Ministry of Health, Labour and Welfare, local governments nationwide established a new “perinatal healthcare system.” Discussions about this system do not imply that midwives should conduct all normal vaginal deliveries. Instead, the focus is on defining roles at various levels, specifying staffing and facility requirements, and establishing a referral system to higher-level institutions [10]. Notably, the perinatal healthcare plan in Osaka Prefecture categorizes low-risk normal vaginal deliveries separately within the system. The guidelines for “response to low-risk pregnancies, deliveries, and normal newborns, including vaginal deliveries” explicitly limit midwifery clinics to managing cases with normal progress while confirming that midwives are authorized to handle normal deliveries [11].

A key aspect of Japan’s perinatal healthcare system is that different medical institutions are designated to manage normal and high-risk cases separately. The system clearly defines the criteria distinguishing normal from abnormal deliveries, outlines the responsibilities at each level, and establishes a transfer system and designated personnel for handling unexpected transitions from normal to abnormal delivery. This system is updated annually and is reflected in the current 2024 edition of the midwifery manual [12].

Thus, the Japanese government leverages midwives as specialized personnel to achieve three main objectives: first, to reduce the medical costs associated with childbirth; second, to address the shortage of obstetricians and ease their workload; and third, to support postpartum care and childcare by providing mental and physical assistance to new mothers.

## Need for amendment of the Medical Service Act in Korea

Japan trains approximately 2,000 midwives annually. As of 2020, 37,940 midwives were actively employed across various settings, including hospitals (64.9%), clinics (21.6%), midwifery clinics (5.7%), and educational and community institutions (7.8%) [13].

In contrast, the number of licensed midwives in Korea declined from 8,266 in 2018 to 8,086 in 2024. Moreover, the number of midwives who completed continuing education and remained active in the field dropped from 499 in 2018 to 410 in 2024. As of 2020, 28.2% of midwives worked in medical institutions, 17% in nonmedical institutions, and 54.8% were inactive. The average age of midwives has steadily increased, reaching 54.6 years—a rise of 8.9 years over the past decade—which places many near retirement age [14].

The aging midwife population in Korea is largely attributable to the very low number of new midwives entering the field. Between 2023 and 2025, only 8, 12, and 9 new midwives were trained, respectively; with 10 midwives receiving training annually at four training hospitals. Beginning in 2025, the inclusion of two additional training hospitals increases this number to 20 midwives per year. Yet, unlike many countries—such as Japan, the United Kingdom, the United States, Australia, and New Zealand—which train midwives in educational institutions, Korea continues to rely solely on training hospitals, rendering its midwifery education system outdated [15]. Furthermore, the gradual disappearance of obstetrics and pediatrics departments in Korean training hospitals exacerbates the severe crisis in developing the future maternity workforce.

To address this problem, it is imperative to revise the Medical Service Act regarding midwifery training. Currently, Article 6 of the Act states that “a holder of a nurse license, who has completed a one-year midwifery training course at a medical institution recognized by the Minister of Health and Welfare,” may be licensed as a midwife upon passing the national examination. This provision should be amended to read: “a person who has completed a midwifery training program at a medical institution or an educational institution.” Although attempts to amend Article 6 have been made since the early 2000s, they were unsuccessful due to opposition. At present, a new legislative proposal is being prepared.

## Establishment of the Midwifery Practice Guidelines and efforts to standardize the directions and quality of midwifery training program

In 2024, the Korean Midwives Association collaborated with professors and clinical midwives to develop standard guidelines aimed at enhancing the quality of midwifery training. Training syllabi were established, and corresponding educational videos are in production. Additionally, the midwifery textbook—designed to align with national examination objectives—is currently undergoing revision to supplement missing content and update existing material based on evidence-based practices, with publication scheduled for the end of April 2025.

## Utilization of midwives: in-hospital midwifery, midwifery clinic, postpartum care, and childcare community projects

In Japan, where 64.9% of midwives were employed in hospitals as of 2020, the government promotes midwifery in two key areas. First, in-hospital midwifery allows midwives to manage normal vaginal deliveries without physician intervention within hospital settings. Second, midwifery clinics enable midwives to oversee prenatal examinations through to postpartum management for normal vaginal deliveries. To support these initiatives, the “Guidelines for In-hospital Midwifery and Outpatient Midwifery Care 2018” were established [16]. Although midwives typically manage low-risk pregnancies, high-risk pregnant women also have the option of selecting midwives for vaginal delivery. Notably, hospitalization fees for childbirth remain identical whether care is provided by physicians or midwives.

Postpartum care has changed in Japan; the traditional custom of staying at a parent’s home after childbirth has largely disappeared. Mothers are typically discharged 5 days after delivery and must manage childcare independently at home. However, the lack of physical and emotional support during the transition to motherhood has been linked to rising rates of postpartum depression. Additionally, increasing newborn deaths associated with maternal neglect or abuse have brought postpartum care to the forefront of social concerns. The rapid rise in child neglect and psychological abuse among mothers caring for newborns alone has prompted the Japanese government to implement comprehensive support for postpartum care since the coronavirus disease 2019 pandemic. Under these circumstances, midwives have emerged as experts in maternal and neonatal care. Local governments sign agreements with branches of the Japanese Midwives Association to entrust maternal care to midwives, who position themselves as reliable birth companions from pregnancy through the postpartum period [17].

In Korea, addressing gaps in the maternity workforce could be partially achieved by actively fostering midwives through the amendment of the Medical Service Act [18] and by initiating pilot projects in in-hospital midwifery, outpatient midwifery care models, postpartum care, and childcare at the local government level, with plans to gradually expand these initiatives nationwide.
